# Chinese Medicine Combined With EGFR-TKIs Prolongs Progression-Free Survival and Overall Survival of Non-small Cell Lung Cancer (NSCLC) Patients Harboring EGFR Mutations, Compared With the Use of TKIs Alone

**DOI:** 10.3389/fpubh.2021.677862

**Published:** 2021-06-18

**Authors:** Yujia Wang, Guoyu Wu, Ru Li, Yingzhe Luo, Xingmei Huang, Lifang He, Huihui Zhong, Shaoquan Xiong

**Affiliations:** ^1^Hospital of Chengdu University of Traditional Chinese Medicine, Chengdu, China; ^2^Department of Traditional Chinese Medicine, Graduate School, Tianjin University of Traditional Chinese Medicine, Tianjin, China

**Keywords:** traditional Chinese medicine, non-small cell lung cancer, progression-free survival, overall survival, cohort study, disease control rate

## Abstract

**Objective:** To explore the efficacy comparison between epidermal growth factor receptor–tyrosine kinase inhibitors (EGFR-TKIs) combined with traditional Chinese medicine (TCM) and single EGFR-TKIs for advanced non-small cell lung cancer (NSCLC).

**Methods:** A total of 91 NSCLC patients with EGFR mutation were divided into an experimental group and a control group (in a ratio of 2:1) to receive TCM and EGFR-TKIs (61 cases) or single EGFR-TKIs (30 cases). Patients in the control group took EGFR-TKIs and those in the experimental group took EGFR-TKIs plus TCM. We analyzed the progression-free survival (PFS), overall survival (OS), disease control rate (DCR), and treatment-related adverse events of two groups.

**Results:** The mPFS of the experimental group and the control group was 12.3 and 8.9 months (*P* = 0.02), respectively, and the mOS of the experimental group and the control group was 28.2 and 24.2 months (*P* = 0.02), respectively. Subgroup analysis showed that for the patients with exon 19 deletion mutation (19DEL), mPFS between experimental group and control group was 12.7 and 10.1 months, respectively (*P* = 0.12). For exon 21 deletion mutation (L858R), the PFS of two groups was 10.8 vs. 8.2 months, respectively (*P* = 0.03). The subgroup analysis also showed that, for the patients with exon 19 deletion mutation, mOS between the experimental group and the control group was 30.3 and 28.7 months, respectively (*P* = 0.19). For exon 21 deletion mutation, the mOS of two groups was 25.5 vs. 21.3 months, respectively (*P* = 0.01). The DCR of the experimental group and the control group was 93.3% and 80.1%, respectively (*P* = 0.77). Grade 3–4 treatment-related adverse events were less common with the experimental group (11.48%) than the control group (26.67%).

**Conclusion:** For NSCLC patients with EGFR mutation, EGFR-TKIs combined with TCM had a certain effect to prolong mPFS and mOS, compared with the use of EGFR-TKIs alone, especially for the patients with L858R. This conclusion has a significant effect on improving the survival of NSCLC patients after EGFR-TKIs resistance. It deserves further study.

## Background

Lung cancer ranks first in the incidence and mortality of malignant tumors in China, accounting for 18% of newly diagnosed cases in 2018, and more than 690,000 people died as a result (1). Lung cancer also has a high morbidity and mortality among all malignancies in the world today (2). According to histological type, it can be divided into small cell lung cancer (SCLC) and non-small cell lung cancer (NSCLC), the latter accounting for about 80–85% (3). Surgical treatment is the radical cure for patients with early- and middle-stage NSCLC in the National Comprehensive Cancer Network (NCCN) guidelines. However, due to the lack of typical clinical manifestations, early screening of lung cancer is difficult. Most of them have diagnosed at advanced stage and have lost the opportunity for surgery. Patients generally have a poor prognosis.

At present, the choice of first-line treatment for advanced NSCLC is based on the presence or absence of specific gene mutations, and epidermal growth factor receptor (EGFR) mutations are the most common gene mutations in Asian NSCLC patients (about 50%) (4). For NSCLC patients with EGFR mutations, the first-line treatment is epidermal growth factor receptor–tyrosine kinase inhibitors (EGFR-TKIs). Compared with chemotherapy, many studies have shown that EGFR-TKIs can significantly improve the progression-free survival (PFS), objective response rate (ORR), and quality of life of patients with advanced NSCLC (5).

However, while patients obtain considerable clinical benefits, the inevitable drug resistance after 9–12 months of medication has greatly limited the clinical application of EGFR-TKIs (6). Therefore, how to enhance the efficacy of EGFR-TKIs and delay drug resistance is a current research focus of advanced NSCLC. In addition to combined chemotherapy, targeted therapy or timely replacement of new-generation TKI preparations (7–9), a number of studies have shown that traditional Chinese medicine (TCM) combined with EGFR-TKIs can improve the quality of life of patients, further prolong patient survival, reduce side effects, and increase curative effect. It also has a definite effect on reversing EGFR-TKIs obtained resistance (10). Therefore, this study aimed to use a cohort study method to analyze the efficacy of TCM combined with EGFR-TKIs in the first-line treatment of advanced NSCLC.

## Materials and Methods

### Diagnostic, Inclusion, and Exclusion Criteria

The diagnostic criteria for advanced lung cancer refer to the Diagnosis and Treatment Specifications for Primary Lung Cancer (2018 edition) (11) and the staging criteria refer to the 8th Edition of the International Lung Cancer TNM Phase formulated by the International Society for Lung Cancer Research (IASLC) in 2015 (12).

Inclusion criteria: (1) Patients older than 18 years. (2) Clinical or pathological diagnosis of stage III B-IV, tissue, body fluid, or cytological genetic test showing exon 19 deletion mutation or exon 21 point deletion mutation (also known as 19DEL and L858R, respectively). (3) Eastern Cooperative Oncology Group Performance Status (ECOG-PS) 0–3 points. (4) Patients expected survival time is >3 months. (5) All enrolled patients signed an informed consent form.

Exclusion criteria: (1) Patients with clinically significant cardiac dysfunction, renal dysfunction, hepatic dysfunction, active infection, or neurologic or psychiatric disorders. (2) Patients administered Chinese medicine for <3 months or if the interval is prolonged to 1 week. (3) The patients were also participating in any other pharmaceutical research. (4) Patients lost to follow-up.

### Study Design

This is a cohort study that included a total of 91 patients with NSCLC. Eligible patients were recruited from First Hospital Affiliated Hospital of Chongqing Medical University and Hospital of Chengdu University of Traditional Chinese Medicine. Patients were divided into an experimental group and a control group (in a ratio of two to one), to receive TCM and EGFR-TKIs (61 cases) or single EGFR-TKIs (30 cases). Patients in the control group were administered EGFR-TKIs and the experimental group were administered EGFR-TKIs plus TCM. Patients in both groups were followed up in outpatient clinics or by telephone every month, and CT (Computed Tomography) or MRI (Magnetic Resonance Imaging) was checked every 3 months to evaluate the curative effect. The patients were followed up until death or the end of clinical trial.

### Drug Administration

Patients in the control group were administered EGFR-TKIs (gefitinib 250 mg/days, erlotinib 150 mg/days, or icotinib 125 mg tid). Patients in the experimental group were given TCM orally on the basis of the same dosage of EGFR-TKIs, one prescription was decocted in water and administered three times a day, 30–50 ml each time.

The basic composition of TCM includes the following: *Huangqi (Astragalus)* (60 g), *Ginseng* (20 g), and *Rhizoma atractylodis macrocephalae* (15 g). For cough symptoms, add *apricot kernel* (10 g) and *Platycodon grandiflorus* (10 g). For symptoms of excessive phlegm, add *Rhizoma pinellinae praeparata* (15 g) and *Dried tangerine peel* (10 g). For symptoms of dizziness and shortness of breath, add *Schisandra seed* (10 g) and *white hyacinth bean* (20 g). For symptoms of diarrhea, add *Rhizoma coptidis* (5 g). For symptoms of night sweats, add *Ophiopogon japonicus* (20 g) and *Radix adenophora* (15 g). For symptoms of rash, add *Honeysuckle* (15 g), *Forsythia* (15 g), and *Cortex Phellodendri* (15 g).

### Evaluation Index

The main observation index is PFS, which is defined as the time from the start of treatment to the first tumor progression or death of the patient due to any reason. The secondary endpoints include overall survival (OS), disease control rate (DCR), and adverse drug reactions. OS is from the time the patient was enrolled to death by any cause or the time of last follow-up. DCR is defined as complete response (CR) + partial response (PR) + stable disease (SD), and tumor progression is evaluated according to the evaluation standards of the WHO.

### Follow-Up

Follow-up in the outpatient clinic or telephone every 2–4 weeks, and check CT or MRI every 2 months to evaluate the disease control rate until death or the time of the last follow-up. Patients who did not attend the outpatient clinic or could not be contacted by phone more than three times (no answer, shutdown, or refusal to answer) were considered as lost cases. The follow-up period started from the first patient enrolled on January 17, 2016, and the last follow-up time was January 31, 2019. The total follow-up time was 36 months. The median follow-up time was 26.2 months (23.5–28.9 months).

### Statistical Analysis

Statistical analysis was performed using SPSS21.1 statistical software: baseline data were analyzed by *χ*^2^ test, curative effect analysis was performed by rank sum test, PFS risk ratio was used for Cox regression model, PFS and OS were tested by Kaplan–Meier and Logrank, and GraphPadPrism6 was used to draw survival curve. *P* < 0.05 was considered statistically significant.

## Results

### Characteristics of Study Participants

From January 2016 to January 2019, 109 patients with NSCLC-sensitive EGFR mutation were included in the study. Among them, three patients were excluded because they did not meet the criteria, five patients were excluded because they were unwilling to participate in the trial, and three patients were excluded due to personal reasons. Finally, they were randomly divided into experimental group (65 cases) and control group (33 cases) according to the ratio of 2:1. During the follow-up period, five cases were lost in the experimental group, one case was lost, and one case was withdrawn due to severe allergic reaction in the control group. There was no significant difference in age, gender, mutation site, clinical stage, PS score, targeted drugs, and brain metastases between the two groups, as shown in Table 1 (*P* > 0.05).

**Table 1:** Characteristics of patients [*n* (%)].

**Characteristics**	**Experimental group (*n =* 60)**	**Control group (*n =* 31)**	***χ*^**2**^**	***P***
**Sex**				
Male	27 (45.0%)	11 (35.5%)	0.761^a^	0.38
Female	33 (55.0%)	20 (64.5%)		
**Age**				
<60 years	24 (33.3%)	12 (38.7%)	0.014^a^	0.90
≥60 years	36 (66.7%)	19 (61.3%)		
**Gene inspection**				
Exon 19 deletion	38 (63.3%)	21 (67.7%)	0.174^a^	0.67
Exon 21 deletion	22 (36.7%)	10 (32.3%)		
**Clinical stage**				
Stage IIIB	9 (15.0%)	5 (16.1%)	0.020^a^	0.88
Stage IV	51 (85.0%)	26 (83.9%)		
**PS Score**				
0–2 points	52 (86.7%)	26 (83.9%)	0.130^a^	0.72
3 points	8 (13.3%)	5 (16.1%)		
**Targeted therapy**				
Gefitinib	53 (88.3%)	25 (80.6%)	1.060^a^	0.60
Erlotinib	4 (6.7%)	3 (9.7%)		
Icotinib	3 (5.0%)	3 (9.7%)		
**Brain metastases**				
Exist	11 (18.3%)	5 (16.1%)	0.069^a^	0.79
Not exist	49 (81.7%)	26 (83.9%)		

a *Represents the *χ*^2^ value for the chi-square test*.

### mPFS

**Figure 1:**
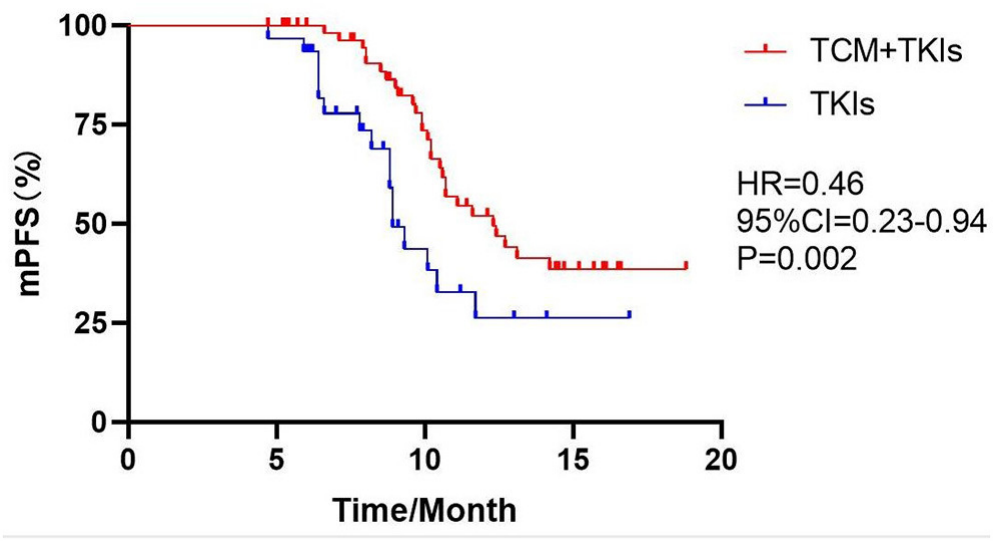
Comparison of mPFS by two groups.

The mPFS of the experimental group treated with EGFR-TKIs and TCM was 12.3 months, which was 3.4 months longer than the 8.9 months mPFS of the control group only using EGFR-TKIs [Figure 1, hazard ratio (HR) = 0.46, 95% CI = 0.23–0.94, *P* = 0.002].

#### Subgroup Analysis of mPFS

**Figure 2:**
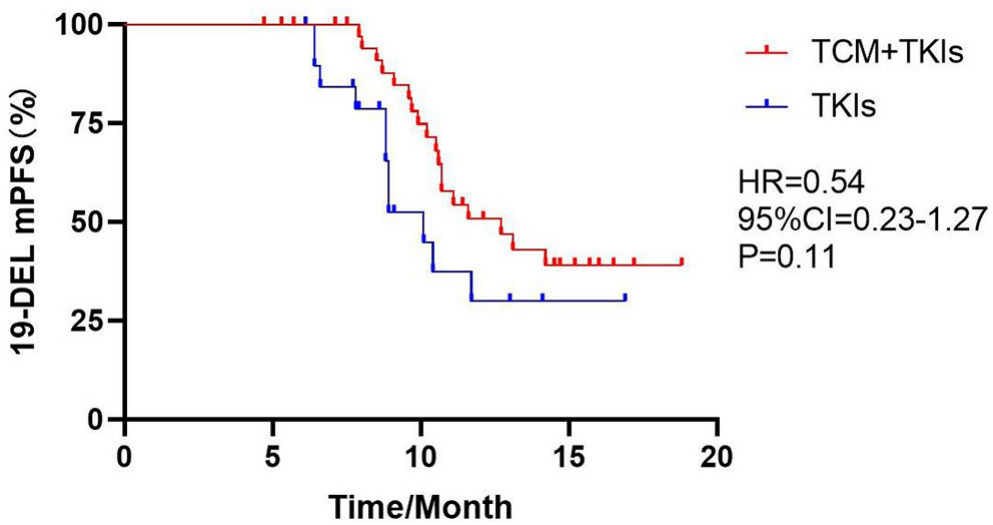
Comparison of 19DEL mPFS by two groups.

**Figure 3:**
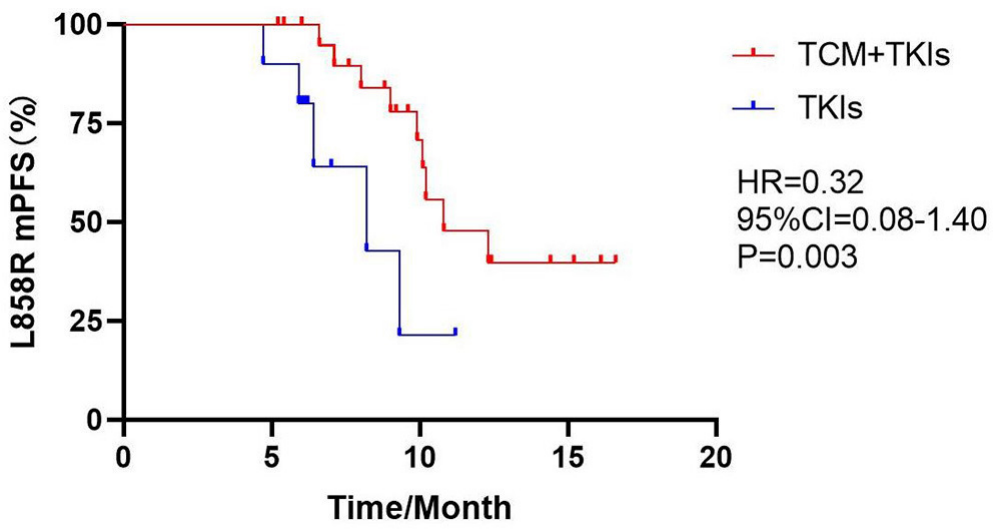
Comparison of L858R mPFS by two groups.

For 19DEL patients, the mPFS of the experimental group and the control group were 12.7 and 10.1 months, respectively (Figure 2, HR = 0.54, 95% CI = 0.23–1.27, *P* = 0.11). For L858R patients, the mPFS of the experimental group was 10.8 months, which was significantly better than the control group at 8.2 months (Figure 3, HR = 0.32, 95% CI = 0.08–1.40, *P* = 0.003).

### mOS

**Figure 4:**
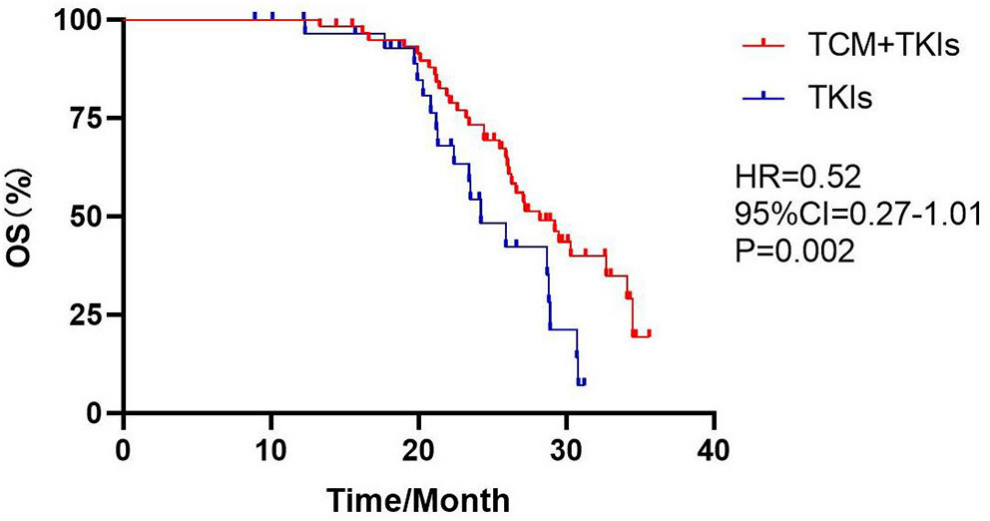
Comparison of mOS by two groups.

The mOS of the experimental group and the control group were 28.2 and 24.2 months, with statistical differences (Figure 4, HR = 0.52, 95% CI = 0.27–1.01, *P* = 0.002).

#### Subgroup Analysis of mOS

**Figure 5:**
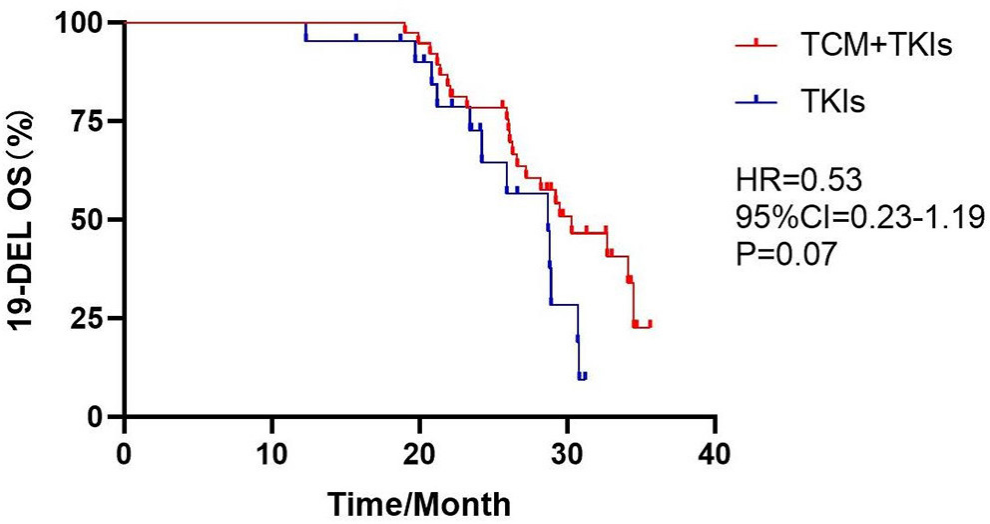
Comparison of 19DEL mOS by two groups.

**Figure 6:**
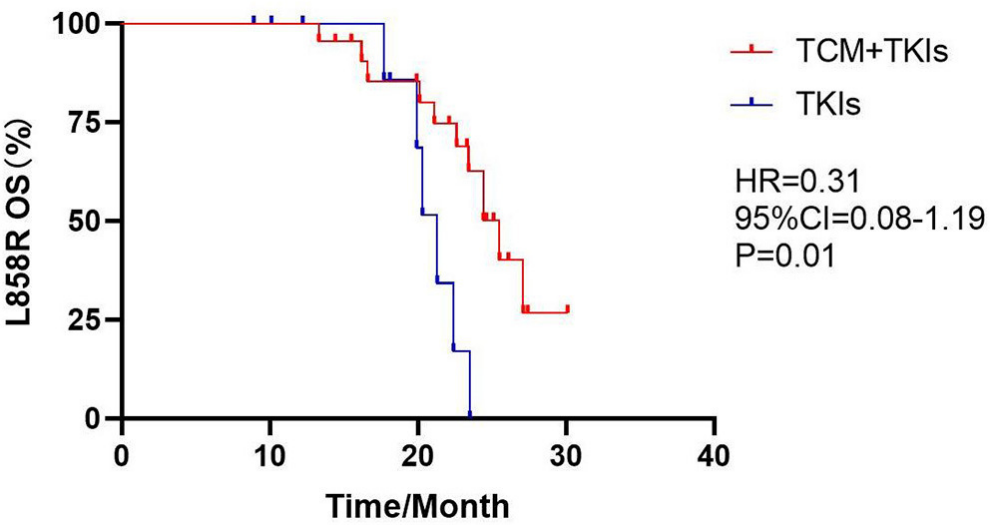
Comparison of L858R mOS by two groups.

For 19DEL patients, the mOS of the experimental group was 30.3 months and that of the control group was 28.7 months; there was no statistical difference (Figure 5, HR = 0.53, 95% CI = 0.23–1.19, *P* = 0.07). For L858R patients, the mOS of the experimental group was 25.5 months, which was clearly better than the control group of 21.3 months (Figure 6, HR = 0.31, 95% CI = 0.08–1.19, *P* = 0.01).

### DCR

The DCR of the experimental group and the control group was 93.3 and 80.1%, respectively (*P* = 0.18); there was no statistical difference, as shown in Table 2.

**Table 2:** DCR (*n*, %), (*P* = 0.18).

**Group**	**CR**	**PR**	**SD**	**PD**	**DCR**
Experimental group	0 (0%)	38 (63.3%)	18 (30.0%)	4 (6.7%)	56 (93.3%)
Control group	0 (0)	14 (45.1%)	11 (35.5%)	6 (19.4%)	25 (80.1%)

### Drug-Related Adverse Reactions

The incidence of grade 3–4 serious side effects such as skin rash, diarrhea, liver damage, oral ulcers, paronychia, etc. between the two groups was as follows: 11.48% in the experimental group and 26.67% in the control group; there was no statistical difference (*P* = 1), as shown in Table 3.

**Table 3:** Adverse reaction comparison table (*n*, %), (*P* = 1).

	**EGFR-TKIs plus TCM (*****n=*****61)**		**EGFR-TKIs (*****n=*****30)**	
	**Any grade**	**Grade 3–4**	**Any grade**	**Grade 3–4**
Adverse reaction	48 (78.69%)	7 (11.48%)	36 (120%)	8 (26.67%)
Rashes	27 (44.26%)	3 (4.92%)	21 (58.33%)	4 (13.33%)
Diarrhea	11 (18.03%)	3 (4.92%)	6 (20.00%)	3 (16.67%)
ALT/AST increasing	6 (9.84%)	1 (0%)	4 (13.33%)	1 (3.33%)
Dental ulcer	4 (6.56%)	0 (0%)	3 (10.00%)	0 (0%)
Paronychia	0 (0%)	0 (0%)	2 (6.67%)	0 (0%)

## Discussion

In China, the number of deaths due to cancer in 2014 was as high as 2.296 million, of which lung cancer accounted for 27.3% (13). Surgical radical treatment is still the first choice for lung cancer, but due to atypical early symptoms, about 70% of patients are diagnosed in the late stage, and the 5-years survival rate tends to be <10% (14). Adjuvant chemotherapy is recommended as a traditional first-line treatment; although its curative effect is objective, it has entered a bottleneck period. With the deepening of the world's tumor molecular biology research, lung cancer targeted therapy has made outstanding achievements, especially the EGFR-TKIs, which has significantly improved the life cycle of lung cancer patients in the past 10 years. According to NCCN Non-small Cell Lung Cancer Clinical Practice Guidelines (Version 1.2021), TKIs are the first-line treatment for patients with EGFR-sensitive mutations. Compared with chemotherapy, TKIs can effectively prolong the PFS of patients, but it cannot be ignored that almost all patients will develop acquired drug resistance after 9–12 months. Therefore, how to enhance the efficacy of EGFR-TKIs and delay drug resistance is an issue to be explored in-depth.

TCM has its unique advantages in the treatment of tumors. It is a unique treatment method with obvious advantages in cancer treatment in China. Chinese medicine has a very early understanding of tumors. As early as 3,500 years ago, there was a record of “tumor” in the oracle bone inscriptions of the Shang Dynasty. With the development of medicine, TCM has changed from the past macro differentiation to the use of modern medical technology to use micro syndrome differentiation to unify prevention and treatment, forming a model of integrated Chinese and Western medicine treatment of tumors with Chinese characteristics. By reviewing many domestic and foreign experimental studies on the treatment of tumors with TCM, we found that TCM can effectively improve the sensitivity of radiotherapy and chemotherapy, minimize the toxic and side effects of radiotherapy and chemotherapy, reduce the recurrence and metastasis of tumor, completely cure the patients with tumors who have received radical treatment, improve the quality of life of patients with advanced tumors, and prolong the survival of patients to achieve “survival with tumors” (15).

In the study of TCM combined with EGFR-TKIs, a number of clinical and basic experiments have confirmed that TCM has the characteristics of multiple pathways, targets, and pharmacological effects, and hard-to-produce drug resistance. It can achieve anti-tumor effects by regulating multiple pathways, effectively reduce the toxic and side effects of TKIs, increase TKIs' sensitivity, and even overcome drug resistance and improve survival benefits (16). Studies have found that many extracts of TCM can reverse the acquired resistance of EGFR: such as astragaloside IV (17), resveratrol (18), curcumin (19), shikonin (20), dihydroartemisinin (21), *Sophora flavescens* (22), *Taxus* (23), Paris saponin (24), etc. Wang et al. through prospective cohort experiments found that the comprehensive treatment program of TCM was not inferior to the maintenance chemotherapy program of modern medicine in prolonging mPFS and mOS in patients with advanced NSCLC. Compared with the control group, the mOS of the treatment group using TCM alone was 75 days longer and showed advantages in terms of low adverse reactions and high quality of life (25). After conducting a cohort study of 60 patients, Zhang et al. found that the PFS of Yiqi Tongluo Jiedu Decoction combined with targeted treatment group was 29.5 months, which was significantly different from that of the single target treatment group, which was 12.4 months (26). Jiang et al. through a cohort study, found that the mPFS of patients in the experimental group treated with Ginsenoside Rg3 combined with Osimertinib was 16 months, which was better than the 13 months of patients in the control group treated with Osimertinib alone (27).

This study collected the clinical data from Chengdu First People's Hospital and Hospital of Chengdu University of Traditional Chinese Medicine to explore the role of TCM on EGFR-TKIs in improving the prognosis of NSCLC patients with EGFR mutations. PFS and OS are important evaluation indicators for the treatment efficacy of advanced lung cancer and also reflect the therapeutic benefit from the aspects of tumor control and OS time. In this study, the mPFS and mOS of patients who used EGFR-TKIs alone were 9.3 and 23.4 months, which were basically consistent with previous studies and related reports (28–30). However, after combined treatment with TCM, the mPFS and mOS of the experimental group were increased to 12.3 and 28.2 months, mPFS has been prolonged by 3.4 months, and mOS has been prolonged by 4 months. Compared with similar studies, with the extension of follow-up time, the mPFS of the study sample did not change much, but the analysis and comparison found that there was a statistical difference in mOS of the two groups of patients, reflecting the efficacy advantage of TCM combined with TKIs. However, it is worth noting that after the disease progression of the enrolled 44 patients, 13 patients stopped EGRF-TKIs treatment and switched to chemotherapy, 15 patients received chemotherapy on the basis of the original EGRF-TKIs treatment, and 10 patients chose to combine other targeted therapies (seven of them used bevacizumab and three used cetuximab), and six patients chose to use third-generation TKIs after detecting the T790M mutation positive, and they were all included in the mOS observation. This may have an impact on the results. Since there seems to be no obvious difference in the proportion of patients between the two groups after changing the treatment plan, and the number of patients is small, data analysis was not performed. However, this trial has continuity and is still under follow-up. In the future, with the gradual increase of sample size, if there are representative results in the follow-up statistics, we will conduct further data analysis. Although some studies have shown that chemotherapy combined with EGFR-TKIs can prolong mPFS by about 5 months than single EGFR-TKIs (31–33), patients in such studies tended to have lower PS scores (0–1 points) and better physical fitness, while patients with better physical condition benefit more from chemotherapy (34); the PS scores of patients included in this study are relatively poor (mostly 2–3 points). It should not be overlooked that although first-line chemotherapy is effective at the initial stage, most patients will progress in 3–4 months (35), and the proportion of patients who can continue to receive second-line chemotherapy is only 30–40% (36), and the efficiency is only about 10% (37). In addition, using EGFR-TKIs combined with chemotherapy and radiotherapy as the first-line treatment will limit the choice of second-line treatment options after tumor progression, while using EGFR-TKIs alone as the first-line treatment, radiotherapy and chemotherapy can also be used as an effective second-line regimen after tumor progression.

The most common EGFR gene mutations in NSCLC are 19DEL and L858R. There is heterogeneity in the predictive effect of different mutation sites for TKIs treatment, and the prognoses of patients with 19DEL and L858R are different. Several studies have shown that although the initial response of EGFR-TKIs to 19DEL NSCLC patients and L858R is similar, the mPFS and mOS of 19DEL patients are significantly higher than those of L858R patients (38–40), and this difference may be attributed to the resistance mechanism. Compared with 19DEL, L858R lung cancer patients have a weaker response to TKI treatment, a worse prognosis, and less survival benefit (mPFS: 9.0 vs. 7.0 months, mOS: 25 vs. 16 months) (41, 42). Relevant studies suggest that patients with exon 21 deletion mutations benefit more when treated with TCM combined with EGFR-TKIs, and mPFS is prolonged by 5 months (14). However, the sample size of this study is larger, and with the extension of follow-up time, the analysis and comparison found that the mOS of the two groups of patients with different mutation sites also has significant differences. For 19DEL patients, the mOS of the control group was 30.3 months, while that of the experimental group was 28.7 months, and the mOS was only prolonged by 1.6 months, with no statistical difference. The reason may be that the insufficient sample size and the low level of evidence-based effect make the difference between the two groups not obvious. For L858R patients, the mOS treated with EGFR-TKIs alone was only 21.3 months, which is basically consistent with the related literature reports (43–45). In this study, the mOS can be extended to 25.5 months after EGFR-TKIs combined with TCM. This suggests that exon 21 deletion mutation patients have greater benefits for mPFS and mOS after combined treatment with TCM in EGFR-TKIs treatment. The result of this research have not been found on domestic and foreign literature websites, and this study is the first article to discover and report this result.

In addition, in this study, the DCR of the control group was 80.1%, and the drug-related adverse reactions were 26.67%. After the combined treatment of TCM, the DCR of the experimental group increased to 93.3%, and the drug-related adverse reactions decreased to 11.48%. Although there was no statistical difference in the disease control rate and drug-related adverse reactions, it also showed that the combination of the two drugs had a tendency to enhance the therapeutic efficacy and reduce the drug side effects.

## Conclusion

In conclusion, the choice of EGFR-TKIs combined with TCM as first-line treatment can achieve longer mPFS than EGFR-TKIs treatment alone and has a clear prolongation of patient survival, especially for exon 21 deletion mutations. Compared with other studies, this study supports the idea that EGFR-TKIs combined with TCM treatment can effectively delay the development of disease and prolong the survival of patients, which can provide more survival possibilities and basis for patients with advanced NSCLC, especially for the patients with exon 21 deletion mutation who have poor efficacy in the first generation of EGFR TKIs. However, there are still some shortcomings in this study. First of all, this study was a cohort study with a low level of evidence base, and there may be some bias affecting the results. For example, the ingredients of TCM water decoction are difficult to stabilize, the number of patients is different between the two groups, the optimal administration time is unknown, etc. Secondly, although this study to some extent proves the effectiveness of TCM in the treatment of advanced NSCLC, the comprehensive treatment of TCM is more complex, the quality control is difficult, and the follow-up time of patients collected in the later stage is shorter. All of these may have some impact on the outcome judgment. In the later stage, we will continue to follow up and strive to obtain longer OS data to continue data analysis to provide more rigorous and high-level evidence for the treatment of advanced NSCLC with Chinese medicine combined with TKIs.

## Data Availability Statement

The raw data supporting the conclusions of this article will be made available by the authors, without undue reservation.

## Ethics Statement

The studies involving human participants were reviewed and approved by Medical Ethics Committee of the Affiliated Hospital of Chengdu University of Traditional Chinese Medicine. The patients/participants provided their written informed consent to participate in this study. Written informed consent was obtained from the individual(s) for the publication of any potentially identifiable images or data included in this article.

## Author Contributions

All authors listed have made a substantial, direct and intellectual contribution to the work, and approved it for publication.

## Conflict of Interest

The authors declare that the research was conducted in the absence of any commercial or financial relationships that could be construed as a potential conflict of interest.
